# Selection of DNA aptamers for ovarian cancer biomarker HE4 using CE-SELEX and high-throughput sequencing

**DOI:** 10.1007/s00216-015-8665-7

**Published:** 2015-04-12

**Authors:** Rachel M. Eaton, Jamie A. Shallcross, Liora E. Mael, Kepler S. Mears, Lisa Minkoff, Delia J. Scoville, Rebecca J. Whelan

**Affiliations:** Department of Chemistry and Biochemistry, Oberlin College, 119 Woodland Street, Oberlin, OH 44074 USA

**Keywords:** Aptamer, CE-SELEX, HE4, Ovarian cancer, Biomarker, High-throughput sequencing

## Abstract

The development of novel affinity probes for cancer biomarkers may enable powerful improvements in analytical methods for detecting and treating cancer. In this report, we describe our use of capillary electrophoresis (CE) as the separation mechanism in the process of selecting DNA aptamers with affinity for the ovarian cancer biomarker HE4. Rather than the conventional use of cloning and sequencing as the last step in the aptamer selection process, we used high-throughput sequencing on an Illumina platform. This data-rich approach, combined with a bioinformatics pipeline based on freely available computational tools, enabled the entirety of the selection process—and not only its endpoint—to be characterized. Affinity probe CE and fluorescence anisotropy assays demonstrate the binding affinity of a set of aptamer candidates identified through this bioinformatics approach.

**Graphical Abstract:**
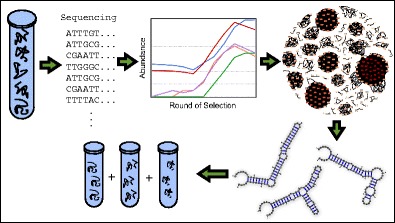
A population of candidate aptamers is sequenced on an Illumina platform, enabling the process by which aptamers are selected over multiple SELEX rounds to be characterized. Bioinformatics tools are used to identify enrichment of selected aptamers and groupings into clusters based on sequence and structural similarity. A subset of sequenced aptamers may be intelligently chosen for in vitro testing.

A population of candidate aptamers is sequenced on an Illumina platform, enabling the process by which aptamers are selected over multiple SELEX rounds to be characterized. Bioinformatics tools are used to identify enrichment of selected aptamers and groupings into clusters based on sequence and structural similarity. A subset of sequenced aptamers may be intelligently chosen for in vitro testing.

## Introduction

The long-term survival of ovarian cancer patients correlates strongly with stage at diagnosis. Local disease, confined to one or both ovaries, responds well to existing treatments, with 5-year survival rates averaging 92 % [1]. By contrast, the 5-year survival rate for patients with metastatic cancer at distant sites is 27 %. These data—and the fact that most ovarian cancers are not diagnosed until metastasis has occurred—provide compelling motivation for the discovery and validation of new ovarian cancer biomarkers that may enable earlier detection.

Using comparative hybridization assays on an array of 21,500 ovarian cDNAs, Hood and coworkers identified the *HE4 (WFDC2)* gene as more highly expressed in ovarian cancer tissue than in noncancerous ovarian epithelium [2]. This observation was supported by serial analysis of gene expression, which also found *HE4* to be amplified in ovarian cancer [3]. A 2003 study demonstrated that serum HE4 protein is detectable via double-determinant immunoassay and is an ovarian cancer biomarker with sensitivity and specificity comparable to that of CA125, the clinical “gold standard,” but with the likely advantage of lower false-positive rates in patients with benign disease [4]. Thorough characterization of protein expression in various tissue types via immunostaining confirmed that normal ovarian epithelium does not express HE4, whereas the protein is strongly expressed on serous and endometrioid tumors, which together constitute the vast majority of ovarian cancer cases [5, 6]. In 2008, the FDA approved the use of a serum HE4 assay for monitoring recurrence in patients with epithelial ovarian cancer. The combination of HE4 and CA125, when used in the Risk of Ovarian Malignancy Algorithm (ROMA) test, is effective at classifying women presenting with a pelvic mass into high- or low-risk categories, which enables the triage of women likely to have ovarian cancer to clinical settings and surgeons with appropriate expertise [7, 8]. The FDA approved this use of ROMA in 2011. Most relevant to the challenge of early detection are the results of ELISA assays performed on banked serum samples collected 1 to 18 years prior to ovarian cancer diagnosis [9]. That study showed the mean concentrations of HE4 (along with serum markers CA125 and mesothelin) in serum samples from cancer patients began to visually increase 3 years before diagnosis, reaching detectable levels 1 year before clinical presentation [9]. Novel analytical approaches to HE4 detection may therefore contribute to early detection of ovarian cancer and associated improvement in patient outcomes.

To complement existing antibody-based detection strategies, many investigators have explored the use of nucleic acid aptamers [10, 11] as affinity probes. Aptamers share with antibodies the property of high-affinity and high-selectivity binding to a target of interest, while having distinct benefits over antibodies in their greater ease of labeling and facile regeneration of native confirmation upon heat cycling. Along with their entirely in vitro development process—the use of animals or cells is not required—these attributes have made aptamers an attractive recognition element employed in a variety of analytical applications [12–14]. Various modes of aptamer selection, referred to as Systematic Evolution of Ligands by EXponential enrichment (SELEX), have been developed [15, 16]. Briefly, a randomized “library” of oligonucleotides is subject to iterative cycles of (1) incubation with the target, (2) separation to resolve bound oligos from unbound, and (3) amplification to reproduce those oligos possessing desired binding attributes. In our development of DNA aptamers with affinity for HE4, we used a capillary electrophoresis (CE)-based separation mechanism [17, 18]. Owing to the high applied field strength used in CE separations, CE-SELEX enables efficient separation of bound and unbound oligos. This selection method has been shown to converge the unselected library onto functional aptamers in fewer rounds than selection methods based on column chromatography or nitrocellulose filtration [19].

Traditionally, the final stage of the aptamer selection process has been to clone the selected oligonucleotide pool into a bacterial expression system, sequence these oligos by Sanger methods, and characterize the affinity of the identified sequences for the target. It has been shown, however, that such approaches can fail to identify high-affinity aptamers [20]. The recent proliferation of high-throughput sequencing (HTS) techniques, also known as next-generation sequencing, deep sequencing, or massively parallel sequencing [21, 22], has made it possible to sequence pools of aptamer candidate oligos with significantly greater coverage than a clone-and-sequence approach. HTS enables the full evolutionary path of the SELEX process to be characterized, not only its endpoint [23]. As a result, better aptamers can be selected with fewer rounds of selection [24], even in a single round [25], by removing the need for the pool to fully converge on a consensus sequence or sequences. Advantages of this approach include reducing time and materials required and minimizing the opportunities for the introduction of polymerase chain reaction artifacts [26] that can bias selection, ultimately causing the loss of high-affinity binders [27]. Similarly, sequences identified by their fold enrichment, rather than raw read counts, can locate high-affinity binders that traditional Sanger sequencing can miss [20]. HTS can also reduce the number of sequences needing to be tested in vitro, when coupled with bioinformatics, by identifying clusters of oligos with a common sequence or structure [28]. A novel method of aptamer affinity determination, MPBind [29], uses a statistical analysis of HTS data to determine aptamer affinity and may prove valuable to users in this field.

Here, we report on our use of CE-SELEX to identify DNA aptamers with affinity for the ovarian cancer marker HE4. Selected DNA was subject to HTS on the Illumina platform. Enrichment and clustering analysis were performed in-house to identify the most promising candidate aptamers for in vitro affinity characterization.

## Experimental

### Reagents

Oligonucleotides—including unselected DNA library, polymerase chain reaction (PCR) and sequencing primers, and labeled aptamers for in vitro testing—were purchased from Integrated DNA Technologies (Coralville, IA). The sequences, previously reported by Bowser and coworkers [23] were forward primer 5′-FAM-AGC AGC ACA GAG GTC AGA TG-3′, reverse primer 5′-biotin-TTC ACG GTA GCA CGC ATA GG-3′, and single-stranded DNA (ssDNA) library 5′-FAM-AGC AGC ACA GAG GTC AGA TG (N)_25_ CCT ATG CGT GCT ACC GTG AA-3′. Nuclease-free water, 25 mM MgCl_2_, 5.0 U/μL Taq polymerase (for PCR), and Blue/Orange 6× loading dye (for gel loading) were purchased from Promega (Madison, WI). Deoxyribonucleotide triphosphates (dNTPs, 10 mM stock) were obtained from QIAGEN, Inc. (Valencia, CA). NuSieve GTG agarose was purchased from Cambrex BioScience (Rockland, ME). 5× Tris Borate EDTA (TBE) was made from TRIZMA Base and boric acid purchased from Sigma (St. Louis, MO), and 0.5 M EDTA (OmniPur; Gibbstown, NJ). The buffer used for aptamer selection and CE separation was 25 mM Tris, 192 mM glycine, 5 mM KH_2_PO_4_, pH 8.3 (TGK) prepared from Thermo Scientific Tris-Glycine powder (Asheville, NC), and KH_2_PO_4_ from Mallinckrodt Chemical Works (St. Louis, MO) using 18.2 MΩ cm water as a diluent. Streptavidin-agarose was purchased from Thermo Scientific Pierce Biotechnology Inc. (Rockford, IL). Bio-Rad columns were purchased from Bio-Rad Technologies (Hercules, CA). Binding and washing (B&W) buffer (10 mM Tris, 2 mM NaCl, 1 mM EDTA at pH 7.6, 2× concentration) was made from NaCl purchased from VWR (Bridgeport, NJ). Absolute ethanol was sourced from AAPER Alcohol and Chemical Co. (Shelbyville, KY). Human recombinant HE4 protein with a glutathione-*S*-transferase purification tag (HE4-GST), GST protein, and the storage buffer for both proteins (50 mM Tris-HCl containing 10 mM reduced glutathione, pH 8.0) were purchased from Abnova (Taipei, Taiwan).

### Capillary electrophoresis and aptamer selection

Capillary electrophoresis aptamer selection was performed on a Beckman Coulter P/ACE MDQ system (Fullerton, CA) with exchangeable UV absorbance and laser-induced fluorescence (LIF) detectors (488 nm excitation, 520 emission). The capillary was 51.3 cm in length and 42.5 cm from inlet to window, with an inner diameter of 50 μm and an outer diameter of 360 μm (Polymicro Technologies Inc., Phoenix, AZ).

Before incubation with target protein, DNA was heated to 95 °C for 3 min and cooled on ice. For each selection round, DNA, target protein, and TGK buffer were combined in 10 μL total volume. The mixture was incubated at 25 °C for 30 min. The equilibrated sample was injected (2 psi for 5 s) and separated (25 kV). LIF detection was used to monitor the separation. During a positive selection round, the eluate was collected into 48 μL TGK buffer until the unbound DNA peak began to elute. In a negative selection round, unbound DNA was also collected into a separate vial containing 48 μL TGK buffer. The injection, separation, and collection process was repeated two more times. The capillary was rinsed with 0.15 M NaOH, water, and TGK buffer between each run. Input DNA concentration was determined by absorbance at 260 nm on a NanoDrop 2000 UV-vis spectrophotometer. For the first round of selection, the unselected library was used, with [DNA] = 10 μM in the incubated sample. Subsequent rounds of selection used DNA collected, amplified, and purified from the previous round as the input DNA. The concentration of input DNA in later selection rounds was 50, 150, 150, 200, 300, and 300 nM, respectively (Table 1).

**Table 1:** Incubation conditions used during rounds of aptamer selection

Round	Pos/neg	Target	[Target]	DNA source	[DNA]
R1	Pos	HE4-GST	50 nM	Library	10 μM
R2+	Pos	HE4-GST	10 nM	R1	50 nM
R2−	Neg	GST	50 nM	R2+	150 nM
R3+	Pos	HE4-GST	5 nM	R2−	150 nM
R3−	Neg	GST	25 nM	R3+	200 nM
R4	Pos	HE4-GST	1 nM	R3−	300 nM
R5	Pos	HE4-GST	0.5 nM	R4	300 nM

### PCR amplification

All PCRs were done using a Mastercycler Personal from Eppendorf AG (Hamburg, Germany). Amplification of selected DNA involved two steps: determination of optimal cycle number and preparative PCR. Master mix was made by combining 484 μL nuclease-free water, 16 μL dNTPs, 20 μL each of forward and reverse primers, 96 μL MgCl_2_, and 160 μL colorless 6× buffer. After mixing, 149.25 μL of the master mix was removed and combined with 0.75 μL Taq polymerase. To 94.5 μL of this completed master mix we added 5.5 μL of DNA collected during selection. This mixed solution was divided equally over thin-walled tubes that were subject to PCR for different numbers of cycles, where each cycle involved three steps: denaturing (95 °C, 30 s), annealing (53 °C, 15 s), and extension (72 °C, 15 s). The samples, which contained different amounts of amplified product, were resolved on a 4 % agarose gel at an applied voltage of 85 V. Gels were imaged on a Kodak Gel Logic 200 Integrated Illumination Cabinet and Imaging System, and photos were digitally improved using Kodak Molecular Imaging software, version 4.5 (Rochester, NY). The fluorescence of forward primers and amplified product enabled visualization of DNA without ethidium bromide staining. The number of cycles that yielded a visible product band with minimal primer and no by-products was used for preparative PCR. In preparative PCR, 646.75 μL of master mix was combined with 3.25 μL Taq polymerase. Seventy microliters of this completed master mix was combined with 5.5 μL of collected DNA in eight separate vials. Completed master mix without added DNA was run as a negative control. After completing the optimal number of PCR cycles, 10 μL of each sample and the control were visualized on a 4 % agarose gel to confirm yield and purity. Remaining samples were pooled and subject to single stranding.

### Single stranding

Double-stranded PCR product was converted to single-stranded DNA using streptavidin columns. For the single-stranding process, 300 μL of streptavidin-agarose slurry was placed in a Bio-Rad chromatography column and washed five times with 500-μL portions of 2× B&W buffer. Pooled PCR product was loaded onto the column with an equal volume of 2× B&W buffer, and the mixture was allowed to incubate at room temperature with gentle vortexing every 5 min for 30 min. The column was then washed ten times, with the following buffers, in order of decreasing ionic strength: four washes with 550 μL 2× B&W buffer; five washes with 550 μL 1× B&W buffer; and one wash with 500 μL ultrapure H_2_O. Thirty micromoles of NaOH (200 μL of 0.150 M NaOH) was then added to the column, gently vortexed, and incubated at 37 °C for 10 min to denature double-stranded DNA (dsDNA). The column was then gently vortexed and the unretained ssDNA (containing the FAM forward primer) eluted into 30 μmol of acetic acid (200 μL of 0.15 M acetic acid) to neutralize the hydroxide. The solution was buffered by the addition of 40 μL of 3 M sodium acetate, followed by 1000 μL of cold 100 % ethanol to precipitate ssDNA. The NaOH elution process was repeated into a separate collection tube, and the two samples (containing ssDNA in ethanol) were incubated at −20 °C or on wet ice for at least 2 h but not more than 12 h. The two portions of eluted DNA were centrifuged at 13,200 RPM for 45 min at 4 °C. Supernatant was pipetted from each tube, leaving 100 μL DNA-containing solution. One milliliter of cold 70 % ethanol was then added to both tubes. After 20 min of spinning at 4 °C, supernatant was removed, leaving 50 μL. The cold ethanol washing process was repeated; after centrifugation, supernatant was carefully removed, leaving 25 μL. Both portions of eluted DNA were then dried in a Speedvac at medium heat for 10 min, followed by 5 min spinning at room temperature. Each tube was then reconstituted in 15 μL of TGK buffer. The DNA was then combined and divided as follows: 10 μL was archived for sequencing, 10 μL was archived for NanoDrop and bulk affinity measurements, and 10 μL was used for the next round of selection.

### Sequencing and bioinformatics

After aptamer selection was complete, DNA collected from each round was amplified using Illumina sequencing primers. Archived DNA from each round was diluted to 100 nM using ultrapure water. Each sample was assigned a unique reverse primer containing the index used for barcoding. Master mix containing all PCR reagents except Taq polymerase and the reverse primers was made from 563 μL nuclease-free water, 18 μL dNTPs, 22 μL forward primer, 108 μL MgCl_2_, and 180 μL colorless buffer. To 97 μL of this mix was added 0.5 μL of Taq polymerase and 2.4 μL of the specific reverse primer. Seventy-four microliters of this mixed solution was added to 1 μL DNA solution; samples and controls were amplified by PCR using an optimized cycle number. PCR products were imaged on a 3 % agarose gel containing 1 μg/mL ethidium bromide to confirm yield and the absence of contaminants or by-products. Samples were sequenced at the University of Wisconsin Biotechnology Center DNA Sequencing Facility.

Bioinformatic screening of the sequenced DNA used a data pipeline based on freely available software, with the exception of enrichment analysis, which used a Python program written in-house. This program (enrichment.py) has been made available on GitHub at https://github.com/rebeccawhelan/PythonEnrichment. After a preliminary analysis of the FastQC files to ensure the sequencing was successful, data were sent into a Biopieces pipeline. Each round’s data was individually read into the pipeline using read_fastqc. Selection primers were removed with remove_primers, using a 5 % mismatch tolerance and a 0 % tolerance for insertions and deletions. All bases beyond the reverse sequence primer (i.e., adaptors and sequencing primers) were removed in this step. These sequences were then filtered using grab to select only sequences with a length of 25 ± 2. These data were processed with uniq_seq, creating one record for each sequence with associated count information. The records were sorted by read count in descending order using sort_records and written to a file as tabular data. From there, the processed data were taken through enrichment analysis, a novel program that determines the fold enrichment for sequences across rounds of selection. Random regions only (with primers excluded) were used in the enrichment analysis to simplify computation; with respect to the enrichment over selection rounds, the primer information is redundant, being identical across all sequences. Fold enrichment has been shown to be a more reliable indicator of binding affinity than read counts [27]. Using CD-HIT-EST [30], the top 1000 most enriched sequences from each round were clustered by sequence homology to determine possible emergent motifs. Sequences were clustered with their primers attached to a sequence identity threshold of 0.8 and assigned to clusters by the highest identity across all clusters.

### Affinity probe capillary electrophoresis

Affinity probe capillary electrophoresis affinity assays were performed using a Beckman P/ACE MDQ (Beckman Coulter, Fullerton, CA) equipped with an argon-ion laser. An unmodified fused silica capillary (Polymicro Technologies, Phoenix, AZ; ID = 50 μm, OD = 360 μm, total length = 49.5 cm, length from inlet to detector = 39.6 cm) was held at 25 °C. Samples were injected from the outlet end, and negative polarity was applied to minimize the distance from injection to detection (length to detector = 9.9 cm). Each sample contained 10 nM FAM-labeled aptamer (synthesized as a 25mer sequence without primer regions), 20 nM fluorescein (internal standard), and 1 mg/mL BSA. TGK was used both as the diluent in sample preparation and as the electrophoresis buffer. To prepare samples, a bulk solution of aptamer in TGK was heated to 90 °C for 3 min, and then put on ice to cool. Fluorescein and bovine serum albumin (BSA) were then added, and the solution was distributed over an appropriate number of sample tubes. Finally, protein (HE4-GST or GST, in separate experiments) was added to a final concentration ranging from 0 to 240 nM. The volume of protein plus protein buffer was constant in all samples. Pressure injection (0.3 psi, 5 s) was used to introduce the sample onto the capillary; separation was achieved by the application (in negative polarity) of 30 kV. Run time was 3 min. The fluorescence was excited at 488 nm and detected at 520 nm. Peak heights were determined by the instrument control software (32 Karat). The change in the size of the free DNA aptamer peak, relative to the internal standard, was used to indicate the complex formation between aptamer and protein. Data were fit with an isotherm equation:

$$ \mathrm{Ratioed}\kern0.3em \mathrm{peak}\kern0.3em \mathrm{height}=\frac{\mathrm{constant}}{\left(1+\left({K}_{\mathrm{d}}/\left(T+0.5\times \left(A+T+{K}_{\mathrm{d}}-{\left({\left(A+T+{K}_{\mathrm{d}}\right)}^2-4\times A\times T\right)}^{0.5}\right)\right)\right)\right)} $$

where *T* is protein concentration (varied) and *A* is aptamer concentration (constant, typically 10 nM), using IgorPro (v. 6.12) graphing software.

### Fluorescence anisotropy

Fluorescence anisotropy was measured using a SpectraMax M5 multimode plate reader with polarizing optics (Molecular Devices, Sunnyvale, CA). Tested aptamers were ordered from Integrated DNA Technologies, Inc. (Coralville, IA) with a 5′ TEX615 (Texas Red) fluorophore. One hundred nanomolar DNA aptamer in buffer (TGK) was heated to 95 °C for 3 min, cooled to 4 °C, then allowed to warm to room temperature. Heat-cycled DNA solution was combined with HE4-GST at a range of final concentrations from 0 to 750 nM in the presence of 0.1 mg/mL bovine serum albumin (BSA). The volume of protein plus protein buffer was constant across all samples. After incubating for at least 30 min in the dark at 25 °C, samples were loaded in duplicate (70 μL/well) into a 96-well Fluotrac 200 black immunology plate (USA Scientific, Ocala, FL) and analyzed in the SpectraMax, with temperature held at 25 °C. The *λ*_ex_ for fluorescence anisotropy was 585 nm, *λ*_em_ was 635 nm, and the wavelength cut-off was 610 nm. Raw data (fluorescence emission parallel and perpendicular to the exCitation) were blank-corrected before the anisotropy values were calculated. Measurements were run at least in duplicate and fit with an isotherm function:

$$ r-{r}_0=\frac{\mathrm{constant}}{\left(1+\left({K}_{\mathrm{d}}/\left(T+0.5\times \left(A+T+{K}_{\mathrm{d}}-{\left({\left(A+T+{K}_{\mathrm{d}}\right)}^2-4\times A\times T\right)}^{0.5}\right)\right)\right)\right)} $$

where *T* is protein concentration (varied), *A* is aptamer concentration (constant), *r* is the anisotropy measured in the presence of protein, and *r*_0_ is the anisotropy in the absence of protein, using IgorPro (v. 6.12) graphing software.

## Results and discussion

An unselected DNA library with *N* = 25 random region was used as the input to the selection process because it provided a good balance of sequence diversity, coverage, and computational tractability. Assuming that each base is equally likely to appear at each position in the random region, there are 4^25^ (~1 × 10^15^) possible sequences in such a library. In our selection, we used 100 pmol (~6 × 10^13^ molecules) of DNA as the initial input, giving any individual sequence an expected abundance of 0.05 (a library with *N* = 23 would give an expected abundance of 1). Using a longer random region would result in lower coverage of sequence space that could result in the loss of useful motifs, whereas a shorter random region might lack the complexity to form relevant secondary and tertiary structures involved in target binding.

**Fig. 1:**
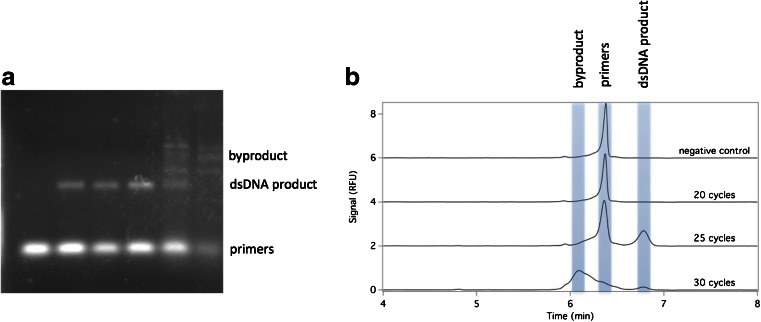
**a** Gel image showing the effect of increasing the number of PCR cycles. **b** Overlaid capillary electropherograms showing the effect of increasing cycle number on PCR product. The traces have been vertically offset for clarity

Table 1 shows the conditions used in our CE-SELEX process. Each incubation condition was completed once, in the order shown (R1 to R5). Positive selection rounds involved incubating DNA oligonucleotides (either unselected library or single-stranded DNA from a previous selection round) with HE4-GST. Nonequilibrium separation conditions were applied to pre-equilibrated mixtures, as is common in capillary-based aptamer selection. At the pH of our electrophoresis buffer, the protein target eluted first, followed by protein-aptamer complexes, and finally the sequences of unbound DNA. We collected solution that eluted during the run and terminated collection before unbound DNA eluted. As received, the protein target of interest (HE4) was covalently attached to a purification tag (GST). To avoid selecting aptamers with affinity for GST, we included two rounds of negative selection, in which GST was the protein target and only the unbound DNA—comprising those oligos without affinity for GST—was collected. DNA collected from each selection round was then amplified by PCR to an optimal number of cycles and single stranded to generate the ssDNA for the next selection round. Figure 1 demonstrates the sensitivity of the PCR product to the number of rounds of PCR conducted. Insufficient rounds of PCR mean that the product of interest is not formed, but too many rounds of PCR result in the consumption of the dsDNA product of interest and the formation of by-products. The sensitivity of PCR when amplifying aptamers has been thoroughly described by Krylov’s group [26]; we include a cycle-determining step in each SELEX round and amplify selected DNA to the optimal cycle number to avoid this problem. In the cycle-determining step, identically prepared samples were subject to different numbers of PCR cycles. These samples were resolved in parallel lanes on an unstained agarose gel and imaged on a UV illuminator through the excitation of the fluorescein primer. The optimal cycle number was that number of PCR cycles that gave a clearly visible band of the desired dsDNA product, but for which no by-products were visible on the gel.

**Fig. 2:**
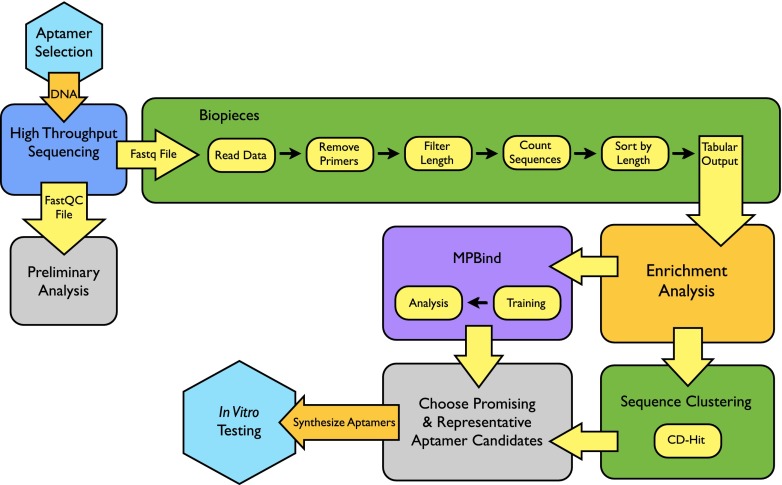
A flow chart showing the steps involved in analyzing high-throughput sequencing data collected after a SELEX experiment

The bioinformatics pipeline used after the completion of the CE-SELEX process is illustrated in Fig. 2. DNA archived after each selection round was sequenced on an Illumina platform. Information about the resulting sequence data is shown in Table 2. The number of oligo sequences yielded by this next-generation sequencing technique is significantly larger (max = 10.1 × 10^6^; min = 2.7 × 10^6^; average = 6.4 × 10^6^) than what would typically be achievable with a clone-and-sequence approach. Sequence duplication, reported as a percent, is the number of duplicate sequences relative to unique. Two trends are notable from the data in Table 2. First, there is duplication of sequences in the—ostensibly random—unselected library (R0). We believe that this phenomenon may result from a combination of errors occurring during synthesis and sequencing. We have observed a strong bias for thymine in ostensibly random libraries (data not shown), suggesting synthesis bias. In addition, the majority of sequence duplication appears to result from contamination by the Illumina sequence adaptor. Second, the percent duplicated sequences increases during the selection process, from around 10 % in the unselected library DNA to around 40 % after the fifth round of selection. We interpret these data to mean that although the unselected library is not composed of a random population of unique DNA sequences—as is often assumed in SELEX—the selection process nonetheless substantially modifies the DNA pool. DNA collected from two rounds of negative selection were also sequenced and served as a negative control in the bioinformatics analysis. Certain sequences that were abundant in the unselected oligo library persisted through multiple rounds of selection. These sequences were abundant in both positive and negative sequence pools, indicating that they are parasitic, rather than truly selected. Sequences with high fold enrichment and cluster abundance were generally not found in negative selection pools; therefore, we were able, through bioinformatics analysis, to filter out these parasitic sequences in silico. MPBind was used to assess possible binding potentials. It was trained using all five rounds of positive selection and used to calculate *Z*-scores for several highly enriched potential aptamers.

**Table 2:** Characteristics of data resulting from Illumina sequencing

SELEX round	DNA sequenced	Number of reads	Sequence duplication
R0	Free	6.7 × 10^6^	11.8 %
R1	Bound	3.8 × 10^6^	13.0 %
R2+	Bound	2.7 × 10^6^	14.0 %
R2−	Bound	8.4 × 10^6^	12.4 %
R3+	Bound	6.4 × 10^6^	14.0 %
R3−	Bound	5.3 × 10^6^	20.0 %
R3−	Free	6.2 × 10^6^	14.0 %
R4	Bound	8.0 × 10^6^	24.1 %
R5	Bound	10.1 × 10^6^	37.7 %

SELEX round numbers are as described in Table 1. The unselected library is designated “R0”

Four promising aptamer sequences were chosen for testing. We chose sequences that were enriched during the selection process and were representative of large clusters. A control sequence was also identified that showed little change in abundance between the unselected pool and the selection rounds. Table 3 shows the sequences that were selected for in vitro characterization. Sequence A1 was the most enriched sequence in R5, and it scores moderately well in sequence-based clustering. It does not appear in any of the negative selection rounds, nor does it have any homologs in the library (defined as having a greater than 85 % sequence identity with a sequence at ≥5 counts in the unselected library). In addition, it scores well in MPBind (*Z*-score = 10.75). Sequence A3 is the third most enriched sequence in R5. Although slightly less enriched than A1, it belongs to the largest sequence cluster. It shares sequence homology with an overrepresented library sequence, but achieving this alignment requires numerous indels and mismatches that are not likely to have resulted from PCR or sequencing error alone. Possibly due to this homology, however, this sequence fairs poorly under MPBind with a *Z*-score of −7.06. Sequence B10 is the second most enriched sequence by R4. It appears in the largest sequence-based cluster in that round, with no library homologs or counts in the negative selection; it has a *Z*-score of −2.08. Unlike the other aptamer sequences tested, B10 is 26 bases long; our bioinformatics analysis was designed to include all sequences of length 25 ± 2. This 26mer sequence is possibly an artifact of PCR or synthesis error, but we chose to study it because of its high degree of enrichment. D3 is the sequence most enriched in R2; it does not appear in the negative selection rounds, and it has a moderately positive *Z*-score (1.6). Finally, L1 was chosen as a negative control. It was the most abundant sequence in the unselected library and subsequent rounds, enabling us to test the hypothesis that a sequence that is initially abundant may still bind to the protein target. However, the sequence of L1 overlaps significantly with the Illumina index adapter, suggesting that it might be a contamination introduced post-SELEX.

**Table 3:** HE4 aptamer candidates chosen for in vitro analysis

ID	Round	Rank	Enrichment	Sequence	*Z*-score	Cluster size
A1	5	1	26	TTATCGTACGACAGTCATCCTACAC	10.75	14
A3	5	3	22	CACAGTGCGTCACATTTAGGGCATT	−7.06	46
B10	4	10	14	CAGTGCGTGCTTATTGGCGTAGCGTC	−2.08	18
D3	2	3	12	ATGGTCGCAAGAACTGAGAATTTAC	1.6	10
L1	0	1	1	CCGTCTTCTGCTTGAAAAAAAAAAA	−15.9	n/a

*n/a* not available

**Fig. 3:**
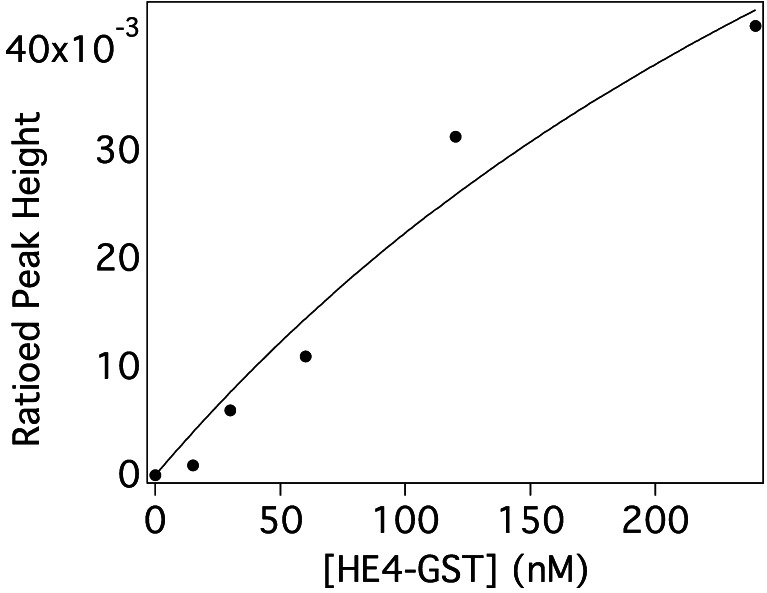
Binding isotherm collected by affinity probe capillary electrophoresis on aptamer A3 and HE4-GST

Two orthogonal techniques—fluorescence anisotropy and affinity probe capillary electrophoresis—were used to characterize the binding of each aptamer candidate for the positive selection protein target (HE4-GST). Figure 3 shows a representative binding isotherm, for the aptamer A3, collected with affinity probe CE, with increasing concentrations of HE-GST. On the *y*-axis is plotted the height of the free DNA peak in the absence of protein relative to the DNA peak in the presence of protein. The data are reasonably well fit by the full form of the binding isotherm equation. Table 4 summarizes the dissociation constant (*K*_d_) values for the interaction of the five aptamer candidates with HE4-GST. All four aptamers selected on the basis of enrichment during selection displayed some affinity for HE4-GST by one or both methods. The sequence D3 chosen from the earliest selection round (R2) displayed the lowest affinity in the anisotropy assay, and no detectable interaction by affinity probe capillary electrophoresis (APCE), suggesting that two selection rounds would not have been sufficient to select a high-affinity aptamer for this particular target. The negative control aptamer, L1, displays discrepant affinity binding, with essentially no binding displayed in the fluorescence anisotropy assay and moderate affinity displayed in the APCE assay. We are currently investigating the cause of this discrepant binding behavior. In a previous study using CE-SELEX and high-throughput sequencing to identify aptamers for rhVEGF_165_, affinity CE and fluorescence anisotropy were used to determine *K*_d_ values [23], yielding estimates of *K*_d_ that differed by up to a factor of 7.3 between the two methods. With the exception of the behavior of L1, our two affinity characterization methods agree to an extent comparable to that seen by other investigators in this area. We also tested the affinity of all five sequences for binding to GST by APCE. All five aptamers tested displayed no affinity for GST, with no positive trend in ratioed peak heights over a concentration range of GST from 0 to 250 nM (data not shown). The negative rounds of selection—in which aptamer candidates displaying affinity for the free GST affinity tag were purged from the DNA pool—seem to have been successful, as indicated by these results.

**Table 4:** Affinity of HE4 aptamer candidates for HE4-GST (*K*_d_) determined by fluorescence anisotropy and affinity probe CE

ID	Fluorescence anisotropy	Affinity probe CE
A1	2.2 μM	390 nM
A3	9.1 μM	500 nM
B10	280 nM	870 nM
D3	26 μM	n/a
L1	>750 μM	300 nM

*n/a* not available

In conclusion, we have demonstrated the proof of concept for using capillary-based aptamer selection, high-throughput sequencing, and a freely available bioinformatics pipeline to select DNA aptamers with affinity for ovarian cancer biomarker HE4. The validity of this combination has also been demonstrated by another very recent publication in this journal [31]. Our current efforts focus on improving the PCR process to reduce the formation of by-products using emulsion PCR [32] and using a six-histidine-modified HE4 as the target protein in place of the more sterically hindered HE4-GST, with the goal of selecting higher affinity aptamers than those reported here. Aptamers with high binding affinity, reflected by low nanomolar *K*_d_ values, are sought for use in bioassays and in eventual clinical application. Inclusion of divalent cations in the selection buffer is also hypothesized to enable greater diversity of secondary structure and therefore greater binding affinity; selection in such a buffer is also in progress.
